# Carriage Rate of Enterobacterales Resistant to Extended-Spectrum Cephalosporins in the Tunisian Population

**DOI:** 10.3390/pathogens13080624

**Published:** 2024-07-26

**Authors:** Ahlem Mahjoub Khachroub, Meriem Souguir, Pierre Châtre, Nour Elhouda Bouhlel, Nadia Jaidane, Antoine Drapeau, Marah El Kantaoui, Sana Azaiez, Jean-Yves Madec, Wejdene Mansour, Marisa Haenni

**Affiliations:** 1 Laboratoire de Recherche Biophysique Métabolique et Pharmacologie Appliquée, LR12ES02, Faculté de Médecine Ibn Al Jazzar Sousse, Université de Sousse, Sousse 4002, Tunisia; ahlem.mahjoub@famso.u-sousse.tn (A.M.K.); meriem.souguir@anses.fr (M.S.); nbouh076@uottawa.ca (N.E.B.); nadia.jaidane@famso.u-sousse.tn (N.J.); marah.elkantaoui@famso.u-sousse.tn (M.E.K.); sana.azaiez@famso.u-sousse.tn (S.A.); wejdene.mansour@famso.u-sousse.tn (W.M.); 2 Unité Antibiorésistance et Virulence Bactériennes, ANSES—Université de Lyon, 69007 Lyon, France; pierre.chatre@anses.fr (P.C.); antoine.drapeau@anses.fr (A.D.); jean-yves.madec@anses.fr (J.-Y.M.)

**Keywords:** ST349, plasmid, F-:A-:B53, Tunisia, ESBL

## Abstract

Enterobacterales resistant to extended-spectrum cephalosporins (ESC) are a marker of the antimicrobial resistance (AMR) burden. They are infecting humans, but the intestinal microbiota can also be transiently colonized without developing symptoms. Healthy carriage can promote silent dissemination of resistant bacteria, and data on this colonization are often lacking. Between 2021 and 2023, a sampling of healthy Tunisian people was carried out. Fecal samples (n = 256) were plated on selective agar, and all collected isolates were characterized by phenotypic (antibiograms) and genomic (whole-genome sequencing) methods. A total of 26 (26/256, 10.2%) isolates were collected, including 24 *Escherichia coli* and 2 *Klebsiella pneumoniae*. In total, 17 isolates (15 *E. coli* and 2 *K. pneumoniae*) presented an ESBL phenotype conferred by the *bla*_CTX-M-15_ gene, and 9 *E. coli* isolates presented an AmpC phenotype conferred by the *bla*_DHA-1_ gene. *K. pneumoniae* belonged to ST1564 and ST313, while *E. coli* belonged to diverse STs including the pandemic ST131 clone. Clonally related ST349 *E. coli* isolates carrying the *bla*_DHA-1_ gene were found in nine individuals. In parallel, four *bla*_CTX-M-15_ -positive *E. coli* isolates carried this ESC-resistance gene on an epidemic plasmid IncF/F-:A-:B53 previously identified in Tunisian pigeons and fish. These findings highlight the spread of genetically diverse ESC-resistant Enterobacterales as well as an epidemic plasmid in Tunisia, emphasizing the need for antimicrobial stewardship to limit the transmission of these resistances in the Tunisian population.

## 1. Introduction

The human gut is inhabited by a large bacterial population organized in a complex community, the microbiota, which ensures crucial functions for human health, including stimulation of the immune system and protection against pathogens among many others [1]. This bacterial community living in the intestine is under the pressure of numerous constraints, among which are the people’s culinary habits and lifestyle. In addition, the intestinal microbiota may also be under pressure from a number of exogenous molecules such as antibiotics. The use of antibiotics generally reduces the overall diversity of the gut microbiota, while increasing both the proportion of enteropathogens and the pool of resistance genes; however, these adverse consequences usually disappear in about 1–1.5 months [2,3]. Interestingly, it was shown that the greater the consumption of antibiotics in a given country, the greater the abundance of resistance genes in the microbiota of its inhabitants, even in individuals who do not consume antibiotics [4].

Antimicrobial resistance (AMR) is a major global health issue; thus, lowering the global AMR burden is of utmost importance. Resistance to extended-spectrum cephalosprins (ESC), mostly mediated by extended-spectrum beta-lactamases (ESBLs) or plasmidic AmpC (such as CMY-2 and DHA-1), is one of the main markers of this AMR burden since ESC-resistant Enterobacterales have disseminated in all domains of the One Health concept. Intestinal colonization of healthy people by ESC-resistant bacteria complicates the understanding of the dissemination pathways since it promotes the silent spread of these resistance determinants. In countries where the AMR burden is low, it has been shown that, after acquisition through hospitalization or travels, carriage of ESC-resistant *Escherichia coli* persist from 3.4 up to 59 months [5,6]. On the contrary, when the proportion of multi-drug-resistant bacteria (MDR) is high in the population, as in Lebanon (60.7%), an important dynamic of loss/acquisition ensures the continuous contamination of a substantial portion of healthy people [7]. A recent systematic review on the trend of ESBL-producing *E. coli* in the community showed that the worldwide intestinal carriage was 16.5% and has been multiplied by 8 over the last 20 years, with considerable disparities between regions (from 6% in Europe to 27.5% in South-West Asia) [8]. This review also pointed out the paucity of data in many regions of the world, notably in Africa.

In Tunisia, two previous studies assessed the proportion of ESBL-producing *E. coli* in the human community. A first study showed that the proportion of ESBL-producing *E. coli* in healthy adult volunteers was 7.3% in 2009–2010 [9], while a second one performed in 2012–2013 showed that 6.6% of the children attending elementary school were ESBL carriers [10]. Both studies reported the predominance of the *bla*_CTX-M-1_ gene in the community, while the *bla*_CTX-M-15_ gene was more widespread in clinical isolates [11,12]. In Tunisian livestock and derived food products, the most frequently reported *bla*_ESBL_ gene was *bla*_CTX-M-1_ [13,14,15], even though the *bla*_CTX-M-15_ gene has also been identified, notably in veal calves and seafood [16,17].

The goal of the present study was to update knowledge on the carriage rate of ESC-resistant Enterobacterales in the Tunisian community and to observe whether the proportion of healthy carriers increased over a ten-year period, as observed at a larger scale by Bezabih et al. in their large metadata analysis [8]. Such studies are particularly relevant in resource-limited countries such as Tunisia where beta-lactam antibiotics are widely used, both in the community and in hospitals. Due to the misuse of antimicrobials in hospitals and the lack of awareness in the community, there is an increase in resistance in the pathogens responsible for nosocomial infections, leading to the use of last-line drugs such as carbapenems and polymyxins. It is, therefore, of considerable interest to identify the potential sources of dissemination of ESC-resistant Enterobacterales, in order to identify the levers of action for lowering the overall burden of AMR.

## 2. Materials and Methods

### 2.1. Ethics

The study was approved by the ethics committee of the Faculty of Medicine of SOUSSE, Sousse University, in accordance with the Helsinki Declaration and informed consent was obtained from all participants

### 2.2. Study Design, Bacterial Isolation and Identification

**Figure 1:**
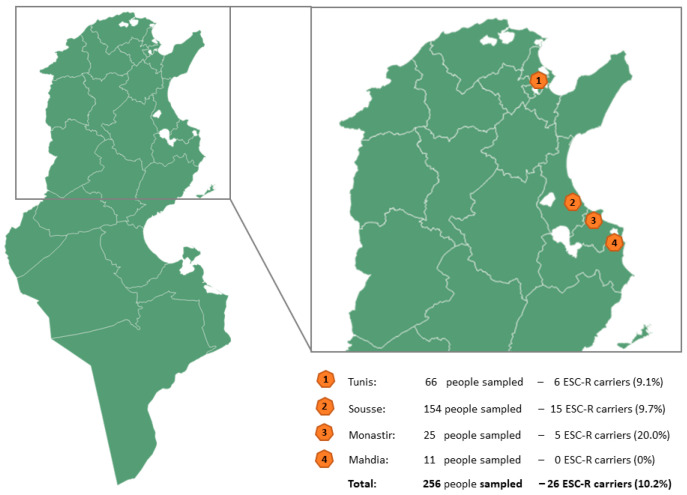
Map of Tunisia with the four cities where healthy volunteers were sampled.

Fecal samples from 256 healthy people recruited from four governorates (Tunis, Sousse, Monastir, and Mahdia) were collected between May 2021 and August 2023 (Figure 1). The calculation of the sampling size was based on the assumption that about 7% of the tested volunteers would be ESC-carriers (mean value of the two previous Tunisian studies on ESC-carriage in healthy people). In order to get a precision of 5% with a 99% confidence level, the minimal sampling size was >240 individuals. The inclusion criteria were (i) age over 18, (ii) subjects with a varied diet, (iii) absence of gastrointestinal disorders, and (iv) no antibiotic therapy for at least one month prior to sampling. All participants completed a questionnaire collecting data about their age, sex, weight, and geographical origin.

Fecal samples (200 mg) were diluted in 5 mL of nutrient broth (Biokar, Tunlab, Tunis, Tunisia) and resistant Enterobacterales were isolated by spreading 100 µL of the dilution on two MacConkey agar plates (Accumix, Tunlab, Tunis, Tunisia), one supplemented with cefotaxime (3 mg/L) and the second one with imipenem (3 mg/L). Selective plates were incubated at 37 °C for 24 h. One presumptive ESC-resistant Enterobacterales colony was arbitrarily selected from each selective plate. Identification of isolates was performed using API20E galleries (bioMérieux, Marcy-l’Étoile, France).

### 2.3. Antibiotic Susceptibility Testing

Antibiograms were performed according to the disc diffusion method on Mueller–Hinton agar, following the guidelines set by the Antibiogram Committee of the French Society for Microbiology (CA-SFM) (https://www.sfm-microbiologie.org/). The *E. coli* ATCC 25922 strain was used as a quality control strain. A total of 16 β-lactam (amoxicillin (20 µg), piperacillin (30 µg), ticarcillin (75 µg), amoxicillin + clavulanic acid (20/10 µg), piperacillin + tazobactam (30–6 µg), ticarcillin + clavulanic acid (75–10 µg), cefalotin (30 µg), cefuroxime (30 µg), cefotaxime (30 µg), ceftiofur (30 µg), ceftazidime (30 µg), cefoxitin, cefepime (30 µg), cefquinome (30 µg), aztreonam (30 µg), and ertapenem (10 µg)) and 14 non-β-lactam (tetracycline (30 µg), kanamycin (30 µg), tobramycin (10 µg), gentamicin (10 µg), amikacin (30 µg), apramycin (30 µg), netilmicin (10 µg), streptomycin (10 µg), florfenicol (30 µg), chloramphenicol (30 µg), sulfonamides (300 µg), trimethoprim (5 µg), nalidixic acid (30 µg), and ciprofloxacin (5 µg)) antibiotics (Mast Diagnostics, Amiens, France) were tested. A double disk synergy test (between amoxicillin + clavulanic acid, ceftiofur, cefquinome and ceftazidime) was used to confirm the presence of ESBLs.

Minimum inhibitory concentrations (MICs) to colistin were performed by broth microdilution according to the EUCAST recommendations [18]. In brief, serial two-fold dilutions of colistin were prepared in 96-well microtiter plates, and a standardized bacterial suspension was added to each well. After incubation, the MIC was recorded as the lowest concentration of colistin (Mast Diagnostics, Amiens, France) preventing visible growth of the bacteria.

### 2.4. Molecular Typing of the Isolates

The detection of the major *E. coli* phylogenetic groups (A, B1, B2, or D) was performed as described by Doumith et al. [19].

### 2.5. Short-Read Whole-Genome Sequencing and Genomic Analyses

DNA was extracted using the NucleoSpin Microbial DNA extraction kit (Macherey-Nagel, Hoerdt, France) according to the manufacturer’s instructions. Library preparation (Nextera XT technology) and sequencing (NovaSeq-6000 instrument) were outsourced (Eurofins, Konstanz, Germany). After sequencing, the reads were quality trimmed (Trimmomatic v0.39) and de novo assembled using Shovill v1.0.4 and the quality of assemblies was assessed using QUAST v5.0.2. Quality control statistics of all sequenced isolates are provided as Supplemental Table S1. Identification was determined using Kraken (https://github.com/DerrickWood/kraken). Online tools from the Cetner for Genomic Epidemiology (CGE; http://www.genomicepidemiology.org/) were used to identify STs according to the Achtman’s scheme (MLSTFinder v2.0.4), as well as the resistance and viruence genes (ResFinder v4.1, VirulenceFinder 2.0.3) and the replicon content and the plasmid subtypes (PlasmidFinder 2.0.1 and pMLST 2.0). SeroTypeFinder 2.0 was used to determine serotypes.

### 2.6. Phylogenetic Analysis

The cgMLST phylogeny was determined through the pyMLST pipeline (https://github.com/bvalot/pyMLST) using the scheme available on the www.cgmlst.org/ncs, which comprised 2513 alleles for *E. coli* (for the matrix distance, see Table S2). The cut-off for highly related strains was <10 allelic differences. The resulting tree was visualized using iTol v6 (http://itol.embl.de/itol.cgi) and high-quality images were obtained using InkScape v1.0.

### 2.7. Long-Read Sequencing

The MinION long-read sequencing libraries were prepared following Oxford Nanopore MinION Technologies (Oxford, UK) protocols, using the native barcoding expansion kit (EXP-NBD104) and the ligation sequencing kit (SQK-LSK109). Sequencing was carried out on a MinION device equipped with a SpotON Mk 1 R9 version flow cell (FLO-MIN106D). Reads from both Illumina and Nanopore were assembled with Unicycler. The resulting contigs were annotated using Bakta (web version 1.7.0/DB: 5.0.0).

### 2.8. Data Availability

The project was deposited in GenBank under the BioProject accession number PRJNA1129310.

## 3. Results

### 3.1. Characterization of Healthy Volunteers and Carriage Rate of ESC-Resistant Enterobacterales

In the study, 256 healthy adults were included, i.e., they presented no infectious disease and had no specific condition that might severely affect their gut microbiota; 99 were selected from the general population and 157 were patients with neurological and neuropsychiatric disorders. Females represented 55% of the study population, and the average age of volunteers was 53 years old (ranging from 18 to 80 years old, with 10% <29 years, 29% from 30 to 49 years, 36% from 50 to 65 years, and 25% from 65 to 80 years. Volunteers originated from the Tunis (n = 66), Sousse (n = 154), Monastir (n = 25), and Mahdia (n = 11) governorates.

Twenty-six of the fecal samples tested (26/256, 10.2%) presented growth on plates containing cefotaxime, while none of them grew on imipenem-containing plates. Of the 26 positive people, 85% were women.

Isolates were identified as *E. coli* (n = 24, 92.3%) and *Klebsiella pneumoniae* (n = 2, 7.7%). *E. coli* isolates belonged to phylogroup D (n = 11), A (n = 8), B1 (n = 3), and B2 (n = 2).

### 3.2. Resistance Phenotypes and Genotypes

**Figure 2:**
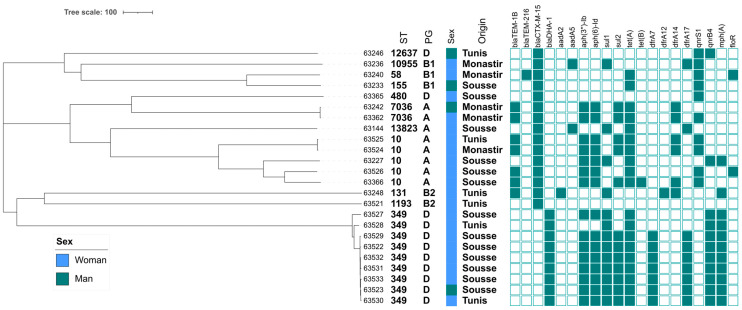
cgMLST-based phylogeny of *E. coli* isolates collected from Tunisian healthy people. Each column corresponds to the ARG listed along the top. A filled box indicates the detection of the gene. ST: sequence types; PG: phylogroups.

Among the 26 collected isolates, 16 (15 *E. coli* and one *K. pneumoniae*) presented an ESBL phenotype, while 9 *E. coli* isolates displayed a pAmpC phenotype (cefoxitin and amoxicillin + clavulanic acid resistance in the absence of a *bla*_ESBL_ gene) and the last *K. pneumoniae* presented both an ESBL and a pAmpC phenotype (Table S3). No isolate was resistant to carbapenems (Table 1). The ESBL phenotype was systematically due to the presence of the *bla*_CTX-M-15_ gene (no other *bla*_CTX-M_ gene was identified), while the pAmpC phenotype was only due to the *bla*_DHA-1_ gene (Table S3, Figure 2).

*E. coli* isolates presented additional resistances to non-beta-lactam antibiotics; the most frequently identified ones were to tetracyclines (75.0%), sulfonamides-trimethoprim (75.0%), and streptomycin (58.3%) (Table 1). Both *K. pneumoniae* were resistant to aminoglycosides (streptomycin, gentamicin), tetracyclines, and sulfonamides-trimethoprim, while the ESBL-producing isolate (#63224) presented additional resistances to kanamycin and tobramycin.

Aminoglycoside resistance was mostly due to the combination of the *aph(6)-Id* (also named *strB*) and *aph(3″)-Ib* genes, respectively, conferring resistance to streptomycin and kanamycin (Figure 2). Resistance to sulfonamides was due to both *sul1* and *sul2* genes, while resistance to trimethoprim was due to *dfrA7* and *dfrA17*. Tetracycline resistance was conferred mainly by the *tet(A)* gene, and quinolone resistance was due to both the *qnrS1* and *qnrB4* genes. Finally, two isolates presented the *floR* gene, conferring resistance to chloramphenicol-florfenicol.

### 3.3. Characterization of ESC-Resistant Enterobacterales

The two *Klebsiella pneumoniae* isolates belonged to the non-pandemic ST1564 and ST313.

A total of 11 different sequence types (STs) were identified over the 24 collected *E. coli* isolates. The most frequently identified ST was ST349 (n = 9/24; 37.5%), followed by ST10 (n = 5; 20.8%) and ST7036 (n = 2; 8.3%) (Figure 2). All other STs were singletons, among which the uropathogenic ST58 and ST131, as well as the zoonotic ST155 were observed.

The allelic distance between isolates from the same ST was further analyzed. Identical clones (<10 allelic differences) were observed for the 2 ST7036 isolates (3 allelic differences), which were collected from 2 people from different families but working in the same restaurant in Monastir. Among the five ST10 isolates, three different serogroups were observed, and the two isolates sharing the same serotype (O19:H19) were also genetically identical (three allelic differences). In this case, no epidemiological link could be found between the two people that even lived in two geographically distant governorates (Tunis and Monastir). For ST349 isolates, 3 serotypes were identified; 2 isolates each presented a unique serotype and were not genetically linked (33–55 allelic differences), while the 7 remaining isolates presented the same O86:H2 serotype and were highly similar (1–4 allelic differences). Among these seven isolates, two were collected from members of the same family in Sousse, two came from people working in the same laboratory but with no other personal contacts, while the remaining five individuals had no epidemiological links.

**Figure 3:**
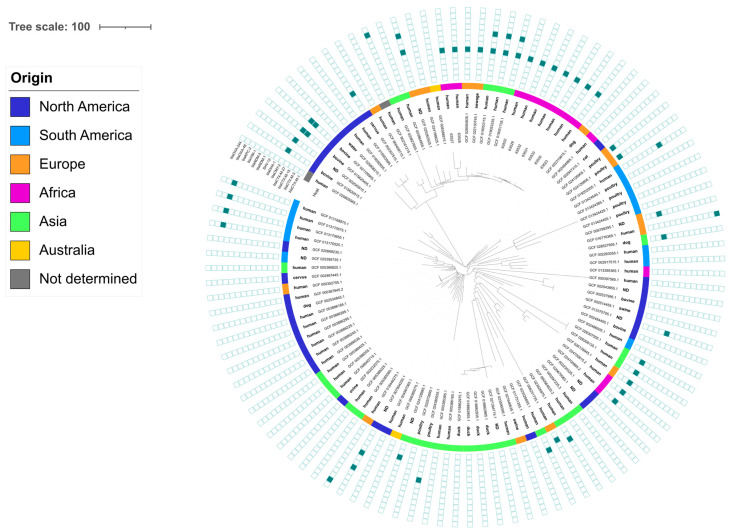
cgMLST-based phylogeny of *E. coli* ST349 isolates. A filled box indicates the detection of that gene.

The high proportion of ST349 isolates coming from epidemiologically independent people prompted us to have a closer look at this sequence type. ST349 isolates are not widespread since only 96 genomes were retrieved from the RefSeq database (Figure 3), which were compared to the Tunisian genomes. Nevertheless, this sequence type was found over all continents, associated with a wide range of genes conferring resistance to extended-spectrum cephalosporins or carbapenems (Figure 3). Among the RefSeq genomes analyzed, five genomes were clustered with the Tunisian ones, and all were *bla*_DHA-1_ carriers.

### 3.4. Characterization of the Genetic Determinants Carrying ESBL/AmpC Genes

Combined analysis of the short-read and long-read sequences revealed that all *bla*_DHA-1_ genes identified in the ST349 *E. coli* were carried on the chromosome. On the contrary, the *bla*_DHA-1_ gene identified in the ST313 *K. pneumoniae* isolate was carried on an IncR plasmid that co-carried the *sul1* and *qnrB4* genes.

In *E. coli* isolates, the *bla*_CTX-M-15_ gene was found on the chromosome in three isolates belonging to ST1193, ST58, and ST155, while the localization of two *bla*_CTX-M-15_ genes could not be determined (#63144 and #63365). The remaining *bla*_CTX-M-15_ genes were identified primarily on IncF plasmids (n = 9), but also on one IncY plasmid and on one IncI1 plasmid (IncI1/pST36-CC3, co-harboring the *bla*_TEM-1B_, *tet(A)*, *aph(6)-Id*, *aph(3″)-Ib*, *floR*, and *qnrS1* genes). This IncI1/pST36 plasmid was large (123,260 pb) and shared 100% identity with other IncI1/pST36 sequences published on the NCBI databases, but with a low coverage (75% with one *E. coli* plasmid collected from a diseased person in Madrid (Genebank OW848982.1) or 72% with one *Salmonella enterica* plasmid collected from chicken meat in Canada (CP016520.1)).

Among the *bla*_CTX−M-15_ genes carried by IncF plasmids, four were harboured by an IncF/F-: A-: B53 plasmid in *E. coli* isolates belonging to ST10 (n = 2) and ST7036 (n = 2). The four isolates carried additional genes (*sul2*, *dfrA14*, *tet(A)*, *aph(6)-Id*, *aph(3″)-Ib*, and *qnrS1*), that were also present on two IncF/F-: A-: B53 plasmids carried by *E. coli* collected from Tunisian pigeons [20]. Two other *E. coli* isolates also displayed an IncF plasmid with an F-:A- backbone, but belonging to the F-:A-:B15 formula. Of note, B15 and B53 alleles differed by eight mutations. The IncF/F-:A-:B15 plasmid from isolate #63246 was 112,180 bp and co-harbored the *qnrS1* and *mph(A)* genes. The seventh isolate presenting an IncF plasmid, #63227, carried an IncF/F2:A-:B- of 83,490 bp, which only co-harbored the *qnrS1* gene. The F2:A-:B- plasmids sharing the closest homology according to the NCBI database were plasmids from *Shigella sonnei* isolated from human stool in Switzerland (CP045525 and CP049186). Finally, the last IncF-carrying plasmid, found in isolate #63248, could not be typed since it presented two IncFII replicons, namely F2 and F51, in addition to the B10 replicon.

In the two *K. pneumoniae* isolates, the *bla*_CTX-M-15_ gene was found on an IncF/F-:A-:B47 in ST1564 and on an IncF_K_/F34:A-:B- in ST313. This large (340,060 bp) plasmid additionally harbored the *sul2*, *dfrA12*, *tetA*, *aph(6)-Id*, *aph(3″)-Ib*, *aadA2*, *qnrS1*, and *mph(A)* resistance genes.

## 4. Discussion

The proportion of ESC-resistant Enterobacterales from healthy volunteers originating from four different geographical regions of Tunisia was 10.2% (26/256). If we only take into account ESC-resistant *E. coli*, for the sake of comparability, an increase in healthy carriage was observed over ten years in Tunisia, from 7.3% in 2009–2010 in adults and 6.6% in 2012–2013 in children, to 9.4% (24/256) in 2021–2023 [9,10]. Our results are coherent with the meta-analysis published by Bezabih et al. [8], which showed an overall proportion of 6% in Europe and 21.4% in Africa. The geographical position of Tunisia on the African continent but with tight links with Europe might explain the intermediate situation observed in our study. Potential explanations for this proportion of healthy carriers are (i) the irrational use of antibiotics in the community, as Tunisia is the second largest consumer of antimicrobials for human use at the world scale [21], (ii) self-prescription or over-the-counter sale of antibiotics, and (iii) lack of optimal knowledge about the rational use of drugs, even among healthcare professionals.

Women represented 85% of the 26 healthy carriers presenting ESC-resistant Enterobacterales, which is similar to a Danish study where a higher proportion of resistance genes was reported in women compared to men (*p* = 0.002) [22]. This difference could be explained by the fact that antibiotics, and particularly cephalosporins and macrolides, are significantly more often prescribed to women than men [23]. This might also be related to the link between the gut microbiota and the endocrine system; indeed, sex hormones such as progesterone may modulate the composition of the microbiota [24]. However, other studies conducted on Chinese (*p* = 0.463) and Spanish (*p* = 0.680) populations did not identify more MDR bacteria in women than men [22]. Consequently, further studies are needed to bring solid scientific evidence. Apart from the gender disequilibrium, an important proportion (6/26, 23.1%) of healthy carriers was overweighted. This is in line with several studies that demonstrated a link between obesity and increased presence of Gram-negative MDR bacteria as well as an enhanced clinical resistome, i.e., a higher frequency and diversity of resistance genes of clinical relevance in the gut microbiota [25,26].

Among the 26 ESC-resistant Enterobacterales, 24 were identified as *E. coli* and two as *K. pneumoniae*. This was expected since *K. pneumoniae* are generally isolated in clinical settings rather than in the community, contrary to *E. coli* which is ubiquitous and the most abundant Gram-negative commensal of the vertebrate gut [27]. An ESBL phenotype was observed for 15 *E. coli* and 2 *K. pneumoniae*, systematically conferred by the presence of the *bla*_CTX-M-15_ gene. This reveals a complete change in the epidemiology of *bla*_CTX-M_ genes in healthy carriers in Tunisia since, among the strains isolated in 2009–2010, all but one (n = 10) presented the *bla*_CTX-M-1_ gene, and the last one presented the *bla*_TEM-52_ gene [9]. Among the eleven carriers described, one was a poultry farmer and a second a veterinarian in the fish industry while all other people had no specific contacts with animals, thus excluding a bias explaining the high proportion of *bla*_CTX-M-1_ genes. Nevertheless, source attribution of the contamination based solely on the CTX-M variant would most likely be erroneous. Indeed, while *bla*_CTX-M-1_ genes are still frequently found in broilers, chicken farms, and meat in general, other genes (including *bla*_CTX-M-15_ but also *bla*_CTX-M-55_ or *bla*_CTX-M-14_ to only name a few) have also been repeatedly identified in animals and food thereof. In strains collected from children in 2012–2013, the *bla*_CTX-M-1_ gene was also dominant (found in 4/7 isolates), while the *bla*_CTX-M-15_ gene was identified in two isolates [10]. The *bla*_CTX-M_ genes have disseminated worldwide, but certain variants are more frequent in specific regions. The three main variants circulating nowadays are *bla*_CTX-M-14,_ *bla*_CTX-M-55,_ and *bla*_CTX-M-15_, with *bla*_CTX-M-15_ being the most widespread on the African continent [28].

In our study, the *bla*_CTX-M-15_ gene was identified as carried by different genetic determinants. First, three *bla*_CTX-M-15_ genes were found on the chromosome, a localization that is increasingly identified, since it allows the stabilization of the gene in the absence of selective pressure [29]. Second, one *bla*_CTX-M-15_ was carried by an IncI1/pST36 plasmid. This plasmid sub-type is uncommon and has been associated to the *bla*_TEM-52_ gene [30,31], but the whole family of IncI1 plasmids has long been associated with an animal source, primarily to broilers [32,33,34,35]. IncI1 plasmids have a high capacity of dissemination and have now been identified worldwide in animals, the environment, and in humans [36,37], including in Tunisia [38]. Third, 10 *bla*_CTX-M-15_ genes were found on IncF plasmids, which are known as major spreaders of the *bla*_CTX-M_ genes [28]. Here, the same plasmid, IncF/F-:A-:B53 was identified in four persons and two genetic backgrounds. This very same formula has already been described twice in Tunisia, in pigeons and in fish [16,20], bringing evidence that this plasmid is emerging as a successful ESBL carrier. Apart from the ESBL phenotype, nine *E. coli* and one *K. pneumoniae* isolates presented an AmpC phenotype due to the *bla*_DHA-1_. This gene is usually found in *K. pneumoniae*, often carried by IncN plasmids; this genetic context was found here in the only DHA-1-producing *K. pneumoniae* identified. On the contrary, the *bla*_DHA-1_ gene was located on the chromosome in all *E. coli* isolates.

Of the 24 *E. coli* isolates, 3 STs were identified in 2 isolates or more: ST10, ST7036, and ST349. ST10 is a ubiquitous and genetically diverse clone that has been reported in animals, humans, and the environment associated with a large diversity of plasmids containing various resistance genes. Two of the five ST10 were clonally related as determined by cgMLST-based phylogeny, but no epidemiological link could be established. The two ST7036 isolates (a very rarely reported ST) were also clonally related but, in this case, they came from two persons who worked in the same restaurant but were otherwise unrelated. Finally, nine isolates of this study belonged to ST349. This clone has been recurrently described in China as a cause of infections in both humans and animals where it harbored the *bla*_NDM-1_ and *mcr-1* genes [39]. It was also found associated to the *bla*_OXA-244_ in the Netherlands [40], and to *bla*_CTX-M-14_ in both chickens and humans in contact in Vietnam [41]. Among the RefSeq genomes analyzed, five genomes clustered with the Tunisian ones, and all were *bla*_DHA-1_ carriers. In total, 3 human isolates from China and 1 sewage isolate from Norway [42] were genetically very close (10–13 allelic differences) to the Tunisian isolates, even though no epidemiological links could be found. Among the nine ST349 isolates from this study, five came from unrelated people while two were collected from members of the same family in Sousse and two others came from people working in the same laboratory. This emphasizes the fact that sharing the same household, but also sharing the same workplace, is a factor of human-to-human dissemination of bacteria, whether resistant or not. This has already been observed between workers in Lebanon [7], where two persons working together but with no other links presented the same ESBL-producing *E. coli*. Intra-household spread has already been described [43,44]. A population-based modelling study also proved that the major source of contamination, which accounts for about two-thirds of the cases, is human-to-human contacts; the last third is largely imputable to food and contacts with farm or domestic animals [45]. In all cases, transmission occurs mainly through dirty hands and surfaces.

One of the main limitations of the study is that we do not have contemporary samples from food, water, and clinical settings, so that the sources of the strains identified in the community cannot be traced. One strain belonging to the typically human-associated ST131 clone was identified in a healthy volunteer in this study, so that human contamination can be hypothesized. But the source of other more ubiquitous isolates such as ST10 or ST155 cannot be inferred. In particular, it would be interesting to have further information on the ST349 clone, which was recurrently found in this study, in people that had no epidemiological links and lived in different regions of Tunisia. This clone, which is widespread according to the available data on the NCBI and that has the capacity to acquire ESBL- or carbapenemase genes, should be monitored in the future. It would also be interesting to monitor the presence of the IncF/F-:A-:B53 plasmid, which seems to become epidemic and has already been found in fish, pigeons, and now humans in Tunisia. Another limitation of this study is the number of volunteers screened. Given the relatively low proportion of healthy carriage of ESC-resistant Enterobacterales in the Tunisian population, the number of isolates that could be retrieved, characterized, and compared to existing data was rather limited. Our results thus open the path to larger-scale studies.

To combat AMR in Tunisia, several steps can be taken based on the findings of this study. First, there should be a concerted effort to improve antibiotic stewardship programs to reduce the irrational use of antibiotics in the community. This would include strict regulations on the sale and prescription of antibiotics, as well as educational campaigns to raise awareness among healthcare professionals and the general public about the dangers of antibiotic overuse and misuse. Second, surveillance systems need to be enhanced to monitor the spread of AMR genes, in the three human, veterinary, and environmental domains. Finally, echoing one limitation of this study, AMR in humans, animals, and food should be addressed using an integrated One Health approach with an ambitious large-scale design.

## 5. Conclusions

Our results showed that 10.2% of the healthy volunteers screened in four different locations in Tunisia were carriers of ESC-resistant Enterobacterales. Identical isolates were found in people sharing the same workplace, but dominant clones and plasmids were also identified in unrelated people. Silent dissemination of resistant bacteria within the healthy population can be promoted by direct contacts or by wider and still unknown pathways This study highlights the urgent need to more efficiently combat AMR in Tunisia, notably outside healthcare settings and in a One Health approach. This includes limiting the uncontrolled use of antibiotics and promoting preventive measures in all sectors.

## Figures and Tables

**Table 1:** Antimicrobial susceptibility phenotypes of all *E. coli* isolates characterized in this study.

	*E. coli* (n = 24)	
	No. of Strains	% of Resistance
Kanamycin	2	8.3
Tobramycin	1	4.2
Gentamicin	1	4.2
Apramycin	0	0.0
Streptomycin	14	58.3
Amikacin	0	0.0
Netilmicin	0	0.0
Tetracycline	18	75.0
Chloramphenicol	2	8.3
Florfenicol	2	8.3
Colistin	0	0.0
Nalidixic acid	2	8.3
Ciprofloxacin	1	4.2
Trimethoprim	18	75.0
Sulfonamides	18	75.0

## Data Availability

All genomic data are publicly available and were deposited in GenBank (PRJNA1129310).

## Supplementary Materials

The following supporting information can be downloaded at: https://www.mdpi.com/article/10.3390/polym16152131/s1: Table S1: Quality controls of all sequenced isolates; Table S2: Distance matrix based on the cgMLST analysis of *E. coli* isolates from this study, as well as ST349 genomes retrieved on the NCBI database; Table S3: Complete characteristics of all isolates from this study.
